# Caveolae-Mediated Endothelial Transcytosis across the Blood-Brain Barrier in Acute Ischemic Stroke

**DOI:** 10.3390/jcm10173795

**Published:** 2021-08-25

**Authors:** Min Zhou, Samuel X. Shi, Ning Liu, Yinghua Jiang, Mardeen S. Karim, Samuel J. Vodovoz, Xiaoying Wang, Boli Zhang, Aaron S. Dumont

**Affiliations:** 1 Department of Traditional Chinese Medicine, Tianjin Medical University General Hospital, Tianjin 300052, China; 2 Clinical Neuroscience Research Center, Department of Neurosurgery, Tulane University School of Medicine, New Orleans, LA 70112, USA; nliu3@tulane.edu (N.L.); yjiang11@tulane.edu (Y.J.); Mkarim2@tulan.edu (M.S.K.); svodovoz@tulane.edu (S.J.V.); xwang51@tulane.edu (X.W.); adumont2@tulane.edu (A.S.D.); 3 Tianjin State Key Laboratory of Modern Chinese Medicine, Tianjin University of Traditional Chinese Medicine, Tianjin 301617, China; zhangboli@tjutcm.edu.cn

**Keywords:** transcytosis, caveolae, blood-brain barrier, ischemic stroke

## Abstract

Blood-brain barrier (BBB) disruption following ischemic stroke (IS) contributes to hemorrhagic transformation, brain edema, increased neural dysfunction, secondary injury, and mortality. Brain endothelial cells form a para and transcellular barrier to most blood-borne solutes via tight junctions (TJs) and rare transcytotic vesicles. The prevailing view attributes the destruction of TJs to the resulting BBB damage following IS. Recent studies define a stepwise impairment of the transcellular barrier followed by the paracellular barrier which accounts for the BBB leakage in IS. The increased endothelial transcytosis that has been proven to be caveolae-mediated, precedes and is independent of TJs disintegration. Thus, our understanding of post stroke BBB deficits needs to be revised. These recent findings could provide a conceptual basis for the development of alternative treatment strategies. Presently, our concept of how BBB endothelial transcytosis develops is incomplete, and treatment options remain limited. This review summarizes the cellular structure and biological classification of endothelial transcytosis at the BBB and reviews related molecular mechanisms. Meanwhile, relevant transcytosis-targeted therapeutic strategies for IS and research entry points are prospected.

## 1. Introduction

Stroke is one of the leading causes of death and disability worldwide, and ischemic stroke accounts for approximately 87% of cases [1]. BBB disruption is a universal underlying factor in reperfusion injury, hemorrhagic transformation, and cerebral edema following the ischemic event. BBB damage results in increased permeability to macromolecules and movement from intracellular to extracellular spaces, consequently increasing intracranial pressure. Severe disruption of the BBB often leads to severe global impairment, seizures, and even death in some patients [2]. However, our understanding of the ultrastructural changes and biochemical pathways, as well as related genomic alterations of BBB disruption following ischemic stroke (IS), continues to evolve.

### 1.1. Prevailing View Was That BBB Leakage in IS Results from Tight Junctions (TJs) Destruction

While the existence of a unique barrier between the central nervous system (CNS) and peripheral circulation was hypothesized by Paul Ehrlich and his student Edwin Goldmann over a century ago [3], the precise anatomical site of the BBB was elucidated only 50 years ago [4,5,6]. Using high-resolution electron microscopy (EM), M. J. Karnovsk et al. [5,6] first observed that subcellular localization of tracers injected into the circulation and found that horseradish peroxidase (HRP) was blocked at TJs between endothelial cells (ECs) of the CNS, confirming the BBB is localized to brain ECs. In contrast, in cardiac and skeletal muscles, and other peripheral tissues, HRP readily permeated through intercellular junctions. Abundant HRP-containing vesicles in ECs indicate that HRP can be transported into peripheral tissue parenchyma via these vesicles. Therefore, the selective permeability of the BBB can be attributed to specialized TJs between CNS ECs. It has been determined that rare vesicles are unique characteristics of CNS ECs, different from peripheral ECs [4,5,6], termed the intercellular barrier and transcellular barrier, respectively. Considering that CNS ECs display remarkably low rates of vesicular transport, research attention has been focused attention on TJs and their proteins. In IS, the biphasic increase of BBB permeability (~6 h and ~48–72 h) after the ischemic event is believed to be due to the disintegration and redistribution of TJs [7,8]. However, it is doubtful that most of the evidence supporting the structural destruction of TJs within 48 h after IS is the consequence of the down-regulation of TJ-related protein expression [9], as there is almost no ultrastructural evidence for TJ disruption under EM observation. A study of embolic stroke in rats showed that BBB breakdown after ictus occurred without ultrastructural evidence of TJ disruption at 25 h after ischemia [10]. In recent years, with the application of in vivo dynamic imaging and in-depth BBB ultrastructure detection, the roles and mechanisms of transcellular transport, or transcytosis, in BBB formation and disruption in acute IS has been discovered and has gradually attracted attention.

### 1.2. Recent Findings Reveal an Increase in Endothelial Transcytosis in the Early Phase after IS Is Independent of TJs

**Figure 1:**
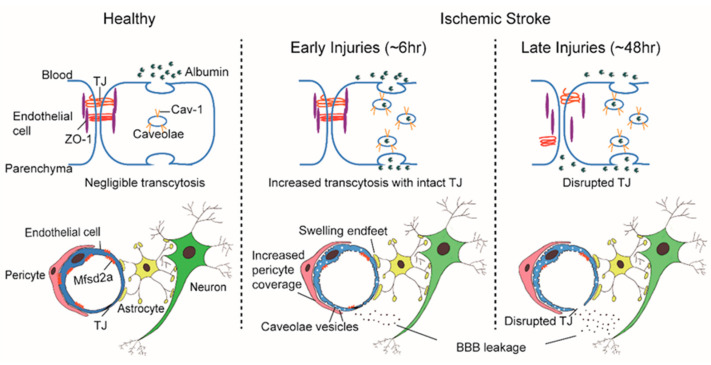
Schematic representation of changes in blood-brain barrier (BBB) integrity and permeability in acute ischemic stroke (IS). In physical conditions, the selective permeability of the BBB is attributed to rare vesicles and negligible transcytosis in central nervous system (CNS) endothelial cells (ECs) and specialized tight junctions (TJ) between ECs. The major facilitator superfamily domain containing 2a (Mfsd2a) is selectively expressed in BBB-containing blood vessels and regulated by pericytes. Mfsd2a establishes a unique lipid environment that inhibits caveolae vesicle formation in CNS ECs to suppress transcytosis and ensure BBB integrity. In IS, the number of endothelial caveolae and caveolae-mediated transcytosis rates increase as early as 6 h after ischemia, macromolecular substances such as albumin can be transported through caveolae-mediated transcytosis, while TJ displayed profound structural defects only after 48 h. The increase of CNS endothelial vesicles is accompanied by a change of pericytes basement membrane coverage and the swelling of astrocytes’ end-feet and their mitochondria.

Employing two-photon microscopy to visualize dynamic TJ changes, Knowland et al. observed fluorescent tracer leakage across the BBB in transgenic mice with fluorescently labeled TJs following induction of the transient middle cerebral artery occlusion (t-MCAO) stroke model. It was noted that BBB leakage began as early as 6 h after ischemia but TJs only displayed profound structural defects after 48 h. Conversely, the number of endothelial caveolae and rate of transcytosis increased beginning at 6 h after ischemia, indicating stepwise impairment of transcellular, followed by the paracellular, barrier mechanisms which contribute to BBB deficits in stroke [11] (Figure 1). Moreover, in an aged mouse model, Nahirney et al. compared the BBB ultrastructure in young adults (3–4 months) and aged mice (18 months) following photothrombotic (PT) stroke, to clarify the structural changes that lead to BBB leakage in aging. At 3 h and 7 h after PT induction, BBB leakage in both ages was associated with a striking increase in endothelial caveolae and vacuoles; while TJs were generally intact, small gaps were detected in a few cases at 72 h after ischemia [12]. These insights to age-associated BBB structural changes suggest that BBB leakage across ages is mediated by increased transcytosis rather than TJ loss [12]. Haley et al. adopted obese *ob/ob* mice paired with t-MCAO as a model of enhanced BBB breakdown and reported microvasculature structural changes of the BBB via two and three-dimensional EM [13]. Vessels from both obese *ob/ob* and *ob/−* control mice showed increased endothelial vesicles at 4 h and 24 h after ischemia, with no significant change in TJ structure or expression of related proteins in isolated microvessels visualized by EM [13]. Compared with *ob/−* controls, *ob/ob* mice had increased lesion volumes and BBB permeability to IgG and HRP. Correspondingly, *ob/ob* mice displayed increased counts of striatal endothelial vesicles at 4 h, localizing at the most severe region of BBB disruption [13]. In light of these reports, we postulate that the presence of endothelial vesicles represents a useful Spatio-temporal index of BBB destruction over the IS disease course.

### 1.3. CNS Endothelial Transcytosis Governs Functional Barrier Formation and Is Gradually Suppressed with Barrier Maturity

Endothelial transcytosis in the CNS is practically negligible under basal conditions, but a prominent feature following acute neurologic injury. Specifically, the rapid increase of this process in IS brings forth the exact role and contribution of endothelial transcytosis in the formation of a sound functional barrier. Delineating this fundamental question in normal development and physiology provides fundamental understanding and key mechanisms dysregulated in stroke. To this end, Chenghua Gu’s team employed a zebrafish model to investigate the involvement of endothelial transcytosis in a vertebrate BBB development. In leveraging this powerful optically transparent model organism with live EM, Gu et al. visualized in real-time the dynamic formation of the zebrafish BBB [14]. Early development of the BBB corresponded with high levels of neural endothelial, a process that is subsequently suppressed later in life. Notably, the timing of suppression of brain endothelial transcytosis coincides with the establishment of BBB function [14]. Similarly, in mice, the same group reported that the formation of the functional blood-retinal barrier (BRB) was similarly conditional on the gradual suppression of brain endothelial transcytosis [15]. Furthermore, because functional TJs were already established on CNS vasculature, the vascular leakage of the immature BRB was attributed exclusively to the process of endothelial transcytosis [15]. Correspondingly, *Mfsd2a^+/+^* and *Mfsd2a*^−/−^ mutant mice, with either increased or decreased transcytosis, mirrored the aforementioned relationship between BRB integrity and transcytosis levels; elevated transcytosis resulted in delayed BRB sealing and mice with suppressed transcytotic activity displayed earlier BRB sealing [15]. Therefore, CNS endothelial transcytosis governs the development of a functional BBB and BRB. The contingent suppression of this process is informed by developmental stage, and time is a principal contributor to the formation of a functional barrier.

Recognizing the normal interplay between transcytosis and barrier formation/integrity alludes to the pathological ramifications when either these conjoined processes are disturbed following IS; the increase in CNS endothelial transcytosis following stroke, and their earlier increase and independence of TJ disruption in the acute phase of IS. Preclinical interventional studies further show that suppression of the BBB endothelial transcytosis in the acute phase of stroke preserves TJ expression, which conserves BBB integrity and brain homeostasis [11]. These insights substantiate a revision of our current paradigm of stroke-induced acute BBB degradation and introduce an exciting new dimension to this major therapeutic target. Still, our concepts and details of how and why transcytosis is initiated in pathological states are incomplete, including delineation of the triggering pathways, what cargo is transported and why, and the disease-specific features of this process across different contexts of acute brain injury. These revelations will substantially enrich our understanding of BBB breakdown, a central hallmark of acute brain injuries, and potentially revolutionize the development of translational drugs stemming the progression of secondary injury by arresting BBB breakdown.

## 2. Cellular Basis of BBB Structure and Endothelial Transcytosis

The remainder of this review briefly summarizes the cellular composition, biological classification, and molecular regulatory mechanisms which underlie BBB endothelial transcytosis. Meanwhile, relevant transcytosis-targeted therapeutic strategies for IS and research entry points are prospected.

The “sandwich” structure of the BBB determines that its constituent components, the ECs, pericytes, and astrocytes, are structurally adjacent and functionally coordinated to control the influx and efflux of substances into the CNS. Pericytes envelop ECs and are embedded within the basement membrane to facilitate extensive and reciprocal intrasignaling. Several influential studies have shown that the precise ratio of pericytes to ECs is critical to BBB integrity; pericytogenesis [16,17,18,19] results in BBB impairment, due to increased endothelial transcytosis. In pathological conditions such as IS, an increase of CNS endothelial vesicles is accompanied by a change of coverage from pericytes at the basement membrane, the swelling of astrocytic end-feet and their mitochondrial dysfunction [12,13] (Figure 1). In young mice, ischemia led to a significant increase in the pericytes process area and vessel coverage over 72 h post ischemia whereas these changes were abrogated with aging. Besides, Caveolae-like vesicles similar to those found in the ECs were also observed in pericytes at 3 and 72 h after ischemia [12]. An influential study found that the physiological blood-brain plasma protein uptake is impaired with age by a shift in transcytosis from ligand-specific receptor-mediated to nonspecific caveolar transcytosis, and this age-related shift in transport occurred alongside a specific loss of pericytes coverage [20]. These findings indicate that pericytes also produce barrier function by regulating endothelial transcytosis; however, the specific genes in pericytes and the paracrine signal mechanisms regulating barrier function between pericytes and ECs remain undefined.

## 3. Biological Classification of BBB Endothelial Transcytosis

Transcytosis is the vesicular trafficking of molecules between the luminal and abluminal cell membranes. Macromolecules are first endocytosed or internalized by vesicles on one side of the cell, trafficked in vesicles, and then exocytosed or released on the other side of the cell. It remains poorly understood what type of vesicles and different transcytosis mechanisms are involved. How vesicle formation and transcytosis are maintained at low levels in brain ECs is also unknown. The current consensus postulates that transcytosis in CNS endothelial cells can be divided into two categories: receptor-mediated transcytosis (RMT), in which ligand-receptor binding mediates endocytosis such as transferrin and insulin [21,22], and nonselective adsorptive transcytosis, in which charged interactions between the molecule and plasma membrane facilitates cargo entry, such as with albumin. Interestingly, a recent study with C57BL/6 mouse revealed decreased plasma protein transport activity through the BBB in the aged brain driven by an age-related shift in transport from ligand-specific receptor-mediated to nonspecific caveolar transcytosis [20].

The two known major endocytic pathways at the BBB are clathrin-mediated and caveolae-mediated [23]. Clathrin-mediated transcytosis is the endocytosis of cargo through clathrin-coated vesicles and is ubiquitous in all cell types [24]. Most RMT is clathrin-mediated, such as transferrin and insulin transport [25]. A review by Xu et al. [25] provides an in-depth description of this complicated process. Prior reports point to the caveolae-mediated mechanism as predominant in CNS endothelial transcytosis after acute IS [11,12,13]. Consequently, IS-induced transcytosis has focused intensely upon alterations of caveolae and relevant proteins. Caveolae are omega-shaped invaginations of the plasma membrane approximately 60–80 nm in length, and are rich in cholesterol and glycosphingolipids [26]. The caveolae were first observed by George Palade et al., who also put forth the notion of caveolae vesicles as mass-carriers of fluid and solutes across the ECs [25]. Subsequent studies have confirmed Palade’s hypothesis, identifying macromolecular substances like albumin, which are transported via caveolae-mediated transcytosis [27]. Conversely, caveolae-deficient ECs showed deficiencies in the uptake and transport of albumin in periphery [28] and CNS [20].

Caveolin and cavin play a key role in the formation of caveolae. Cavin is an adaptive protein that forms oligomers crucial for membrane bending, and caveolin is an integral membrane protein that binds to cytosolic cavin to form small vesicles [29,30]. Caveolin (Cav) can be subdivided into three groups based on their interactive functions with cavin: Cav-1, Cav-2, and Cav-3. The latest data show that Cav-1 and cavin-1 act synergistically to generate a unique lipid environment in caveolae [31]. In the brain, Cav-1 and Cav-2 are mainly expressed in ECs, Cav-3 is expressed in astrocytes, and notably, only Cav-1 is considered to be essential for caveolae formation [32,33].

## 4. Molecular Regulatory Mechanisms of Caveolae-Mediated Transcytosis in IS

### 4.1. Cav-1

Increasingly, studies associate Cav-1 with the permeabilized BBB following various pathological conditions, including IS. It has been found that Cav-1 expression increases in the rat cortical at 12 h after hypothermic brain injury, preceding the decreased expression of occludin and claudin-5 at 48 h [34]. Moreover, Phoneutria nigriventer spider venom (PNV)-induced BBB breakdown and increased vesicle trafficking was related to increased Cav-1α expressions in cerebellar capillaries and Purkinje neurons at 2, 5, and 24 h after brain injury. The major fraction of Cav-1 upregulation was localized to the white matter and granular layers [35]. The role of Cav-1 is highlighted in cortical spreading depolarizations (CSDs), where injury-induced BBB permeability is specifically mediated by increased endothelial caveolae-mediated transcytosis starting at 3~6 h and lasting up to 24 h [36]. Notably, CSD-induced BBB leakage was conspicuously absent in *Cav-1*^−/−^ mice compared with age-matched wild-type (WT) controls [36]. Knowland et al. report that transcellular, but not paracellular, permeability is reduced in the cortical vessels of Cav-1 null mice at 6 and 27 h after t-MCAO. Cav-1 deficiency only significantly reduced the amount of circulating albumin transported into the brain parenchyma with endosome vesicles routed, but no effects on biocytin-TMR or IgG transportation were detected [11]. Moreover, TJ ultrastructural morphology expression levels and subcellular localization of major TJs, claudin-5, occludin, and ZO-1, in healthy brain ECs are indistinguishable between those of *Cav-1^−/−^* mice [11].

These findings endorse the notion that upregulation of Cav-1 is functionally related to an increase in BBB permeability via increased endothelial transcytosis. Given the important role of caveolae and Cav-1 in early BBB breakdown following IS, they are attractive targets of modulation to attenuate BBB dysfunction, and thus theoretically provide the downstream benefits of arresting edema formation and infiltration of peripheral immune cells, crucial elements for improving stroke outcomes. Still, it is worth mentioning that studies have also reported that Cav-1 deficiency can aggravate the injury of IS. Work by Knowland et al. supports the divergent detrimental effects of deficiency, showing increased lesion volumes in *Cav1*^−/−^ mice compared to WT controls [11]. Hirt and colleagues demonstrated a protective role of endogenous Cav-1 in the first week of IS which acted to promote neovascularization, astrogliosis and scar formation [37,38]. Furthermore, increased expression of Cav-1 was found in new blood vessels in the lesion and peri-infarct areas. *Cav1*^−/−^ mice also displayed enhanced hemispheric swelling, lesion volume, and worsened neurological outcomes compared to WT controls. These worsened outcomes coincided with reduced neovascularization and modified astrogliosis, without the formation of proper glial scarring around the lesion at 3 days post injury [37], decreased perivascular AQP4 expression in peri-infarct and contralateral cortical regions, and impaired perivascular AQP4 covering [38], of stroked *Cav1*^−/−^ mice. Gu et al. recently showed that, unlike other vascular segments of ECs in the CNS, arteriolar ECs have abundant caveolae and actively relay signals from the CNS to smooth muscle cells through a caveolae-dependent pathway to realize vasodilation, which is independent of the endothelial NO synthase (eNOS)-mediated NO pathway [39]. In patients, a clinical study suggested that the loss of Cav-1 may be associated with the hemorrhagic transformation following IS. Castellanos M. et al. tested the serum Cav-1 levels of 133 first-time stroke patients that underwent thrombolytic therapy with recombinant tissue plasminogen activator (rt-PA) within 4.5 h after the onset of symptoms were detected. Results showed circulating Cav-1 levels in stroke patients were higher than those of healthy controls, while the basic level of serum Cav-1 in patients with substantive and symptomatic hemorrhage was lower than that in other patients. The level of serum Cav-1 in patients with hemorrhagic transformation remained stable within 72 h after stroke, while the level of serum Cav-1 in other patients decreased during this period, suggesting that a low serum Cav-1 level can be used as an independent predictor of hemorrhagic transformation after r-tPA treatment [40].

The seemingly contradictory results reviewed from these studies indicate the complex pathways which participate in transcytosis, further complicated by disease states. The divergent effects of Cav-1 in stroke point to its pleiotropic capacities, including cell growth, differentiation, cholesterol trafficking, and cellular senescence [41]. The use of different animals, cell models, transgenic animals knocked out or fixed with Cav-1, and large samples of clinical studies, may help to delineate the role of Cav-1 in IS.

### 4.2. Mfsd2a

Significant differences differentiate barrier characteristics and vesicle numbers between central and peripheral ECs; whether BBB ECs express a specific set of genes that govern the formation and maintenance is a major outstanding question. Again, innovative work from the Gu team illuminated answers to this question. Gu et al. identified over 200 highly expressed differential genes in cortical ECs that are barely expressed in lung ECs [42]. Among these differentially expressed genes, the major facilitator superfamily domain containing 2a (Mfsd2a) stands out as selectively expressed in BBB-containing blood vessels and regulated by pericytes [42]. Previously, Mfsd2a was reported to be a transmembrane protein expressed in the placenta and testis, which have highly restrictive barrier properties [43]. Mfsd2a mutants display continuous BBB leakage from embryo to adulthood [42], and EM imaging shows a dramatic increase in CNS ECs transcytosis without obvious TJs abnormality [14,42]. Further studies showed that the lipid transport function of Mfsd2a may lead to a significant difference in lipid signaling between the CNS and peripheral ECs; lipids transported by Mfsd2a establishes a unique lipid environment that inhibits caveolae vesicle formation in CNS ECs to suppress transcytosis and ensure BBB integrity [44,45]. These findings identify Mfsd2a as a key regulator of BBB function that may act by suppressing caveolae-mediated transcytosis in CNS ECs. Based on the reviewed literature, it is our belief that the preclinical experimental data indicate that caveolae-mediated transcytosis is actively inhibited in CNS ECs to ensure BBB integrity, which presents a promising therapeutic target that warrants future study (Figure 1).

### 4.3. VEGF

Increased BBB permeability is correlated to hemorrhagic transformation, which represents the main limitation of rt-PA thrombolytic therapy in IS. Presently, tPA remains the only approved therapeutic treatment available for IS patients; however, strict inclusion criteria and a limited therapeutic time window limit its ubiquitous usage for patients. Creative strategies to overcome these realities would substantially alter the treatment approach for IS and benefit many patients yearly. Suzuki et al. showed that the transient increase of BBB permeability is related to the endothelial transcytosis in the ischemic area, which is partially regulated by vascular endothelial growth factor (VEGF) [46,47]. They found that rt-PA treatment at 4 h after MCAO transiently increased BBB permeability, accompanied by increased endothelial transcytosis in ischemic regions that could be inhibited by low-density lipoprotein receptor family (LDLRs) or VEGF receptor-2 (VEGFR-2) antagonists. In the immortalized bEnd.3 ECs ischemic model, rt-PA upregulated VEGF expression and VEGFR-2 phosphorylation in an LDLR-dependent manner. rt-PA treatment further increased monolayer endocytosis and transcytosis of bEnd.3 ECs, which could also be inhibited by antagonists such as LDLR, VEGF, or VEGFR-2. The authors suggested that rt-PA increases the permeability of BBB in IS by inducing VEGF, which partially mediates the subsequent increase of endothelial transcytosis. Although the investigation is still in an early phase, modulating endothelial transcytosis may improve tPA efficacy or tolerability, and studies inhibiting the production of VEGF may complement thrombolytic therapy of rt-PA after stroke [46]; Interestingly, recent studies have indicated that besides the most well-studied angiogenic growth factors, the guidance molecules also play a role in post stroke BBB leakage [48]. Rust et al. demonstrated that anti-Nogo-A antibodies partially reverse the VEGF-induced BBB leakage when coadministrated through implanted mini-osmotic pumps for seven consecutive days after stroke. Moreover, anti-Nogo-A antibodies have similar proangiogenic effects as local VEGF treatment and do not increase vascular permeability in the peri-infarction regions [49]. Whether their mechanism of action is related to the inhibition of endothelial transcytosis remains unclear. However, this does provide a novel therapeutic strategy, and considering the broad impact of expanding tPA access all promising avenues should be investigated.

## 5. Therapeutic Strategies Targeting Caveolae-Mediated Transcytosis in IS

### 5.1. Transcytosis-Targeted Drugs and Therapies

Various intervention strategies derived from natural medicines or traditional therapies have demonstrated targeted effects on CNS endothelial transcytosis in IS. Ping et al. identified increased numbers of cortical endothelial caveolae at 3 h and 6 d after t-MCAO, accompanied by a corresponding increase in Cav-1 expression. Treatment with compound Cerebralcare Granule^®^ (CG, Tasly Pharmaceutical Co Ltd., Tianjin, China; a compound Chinese medicine composed of eleven herbs) targeted endothelial transcytosis by significantly reducing the number of cortical endothelial caveolae and expression of Cav-1. Targeting this mechanism rescued BBB-integrity after cerebral ischemia in rats [50]. Alternatively, Zhang et al. demonstrated that green tea polyphenols (GTPs, a polyhydroxy phenolic compound with epigallocatechin-3-gallate to be the major and the most effective constituent) can also arrest ischemia disruption of BBB and brain edema, and may confer its neuroprotective effect by down-regulating Cav-1 and phosphorylation of extracellular signal-regulated kinase 1 and 2 (ERK1/2) [51]. Using the compound HC-067047, an antagonist of transient receptor potential vanilloid 4 (TRPV4), Xie et al. improved BBB integrity after cerebral ischemia by reducing the expression of Cav-1 and Cav-2 [52]. Zou et al. found that electroacupuncture at the Baihui Acupoint significantly improved the permeabilized BBB, reduced brain edema, and down-regulated the expression of phosphorylated Cav-1 in ECs [53]. In vitro studies have shown that the representative isoflavone in Astragalus Radix Calycosin and its glycoside form calycosin-7-O-β-d-glucoside can significantly inhibit the decrease of Cav-1 protein in hypoxic and glucose-deprived cerebral microvascular ECs [54]. Results of studies reviewed here suggest great potential and relevance of traditional medicine and therapy as possibly powerful transcytosis-targeted intervention tools. With the promising results of these studies, it would be an interesting future direction to find the most effective active components and match them or to test combination therapies using electroacupuncture therapy, and other modern medicine approaches such as r-tPA, to see if there are synergistic effects.

### 5.2. Transcytosis-Targeted Drug Delivery Strategy

Increasing studies have shown that caveolae-mediated transcytosis increases as an immediate response in the acute stage of IS. This response may be a precursor of irreversible nerve injury, while some scholars have innovatively proposed that it may also serve as a new and promising opportunity to deliver therapeutic drugs into the brain [55,56]. Nanoparticles and albumin conjugates are considered promising drug delivery strategies that may be well suited to leverage this window of enhanced caveolae trafficking into the CNS [56]. Al-Ahmady et al. intravenously injected liposomes into mice following tMCAO and found an early (4 h after tMCAO) and a delayed (48 h after t-MCAO) window of liposome accumulation colocalizing in brain regions of enhanced Cav-1 expression and immunoglobulin penetration. These results indicate that selective liposomal brain accumulation coincides with a biphasic enhancement of transcellular transport, followed by delayed impairment to the paracellular barrier. This process preceded histological evidence of neurological damage in the acute phase and maintained long-term liposomal co-localization within the neurovascular unit, which presents great potential for neuroprotection. Additionally, levels of liposomal uptake by glial cells were similarly selectively enhanced in the ischemic region at 2–3 days after IS, highlighting their potential glial blocking effect to delay inflammatory responses or shift the polarization of microglia/macrophages toward brain repair [57]. Voigt et al. developed a polyanionic lipid nanoparticle that selectively undergoes caveolae-mediated transcytosis in endothelial cells. The principle underlying this technology is the high-affinity hydrophobic interactions that promote the association of the lipid nanoparticle with caveolar lipid rafts, while the negatively charged sulfonate polymer on the nanoparticle surface prevents nonspecific electrostatic interaction with the cell membrane [58]. Albumin is one of the most prominent proteins in the circulation that is trafficked across the damaged BBB by caveolae. After leaking through the BBB, evidence suggests albumin is endocytosed to some degree by astrocytes, neurons and microglia, which could theoretically deliver a drug payload [59]. Albumin is a native carrier of fatty acids and thus is well-suited to be a platform for a variety of conjugations. The proposed strategy for albumin backpacking involves conjugating drugs to fatty acid molecules that noncovalently interact with endogenous albumin following intravenous injection of the target molecule. This approach is complimented by the fact that albumin readily passes through the brain endothelium and is not associated with early endosomes or lysosomal pathways [11,56]. To our knowledge, nanoparticles and albumin-drug conjugates have not yet been translated for the treatment of CNS disorders, but they represent a versatile avenue for localized drug delivery in the early stages of IS and other acute neuronal diseases.

Focused ultrasound (FUS) is a new therapeutic method that can lead to a transient, targeted and reversible opening of the BBB. Deng et al. determined that FUS treatment in combination with microbubble cavitation can enhance BBB permeability through caveolae-mediated transcytosis by upregulating the Cav-1 expression and number. This Cav-1-mediated transcytosis may cooperate with other transport pathways to induce the opening of the BBB [60]. Pandit et al. compared the leakage of substances of different molecular weights in Cav-1-deficient and WT mice exposed to ultrasound. Results showed that the two strains showed a significant difference in the BBB leakage of large molecular substances (500 kDa), but no difference in the leakage of small molecular substances (3 kDa and 70 kDa) was detected, suggesting that Cav-1-related transcytosis induced by therapeutic ultrasound may play a key role in the transport of macromolecules [61]. To date, no original research has reported on the application of ultrasound in the treatment of IS, but these studies have shed light on the mechanism of a transient, targeted, and reversible opening of the BBB for drug delivery.

## 6. Open Questions and Challenges

In all, research on caveolae-mediated BBB endothelial transcytosis in acute IS is on the ascendance. The increase of transcytosis is considered a precursor to the permeabilized BBB in the acute phase of IS but may also represent a new opportunity to deliver therapeutics to the injured brain. The physiological and pathological significance, related regulatory mechanisms, specific targets, and targeted CNS drug delivery all remain to be defined, while clinical translation of this exciting process depends on further preclinical and clinical research. It is already known that caveolae-mediated BBB endothelial transcytosis is a transient and reversible process that does not destroy the essential structure of the BBB. This process is accompanied by the occurrence and change of transcytosis at the BBB endothelium such as the shift with aging from efficient ligand-specific receptor-mediated transcytosis to inefficient nonspecific caveolae transcytosis during blood-brain transport [20]. Recent works also highlight the relationship with sleep function that rapid eye movement (REM) sleep loss and recovery affects transcytosis at the BBB by regulating caveolae formation at brain ECs [62]. However, lack of dynamic and real-time observation limits our further understanding of this process. Questions like: “Do different transcytosis pathways have different triggering/gating mechanisms, transport routes, and different contributions to BBB substance communication under different physiological and pathological conditions?” will need to be answered before we can implement transcytosis-based interventions in disease. An in-depth study of the anatomically specific mechanism of BBB transcytosis may be a breakthrough for future research. In addition, visualization of BBB transcytosis in vivo using imaging techniques and sensitive fluorescent molecules would further elucidate the intercellular signal transduction pathways between pericytes and ECs that inhibit transcytosis. Based on the literature and rationale reviewed here, developing transcytosis holds promise for not only providing new strategies for the treatment of IS and other CNS diseases, but also provides new opportunities for central drug delivery.
